# Lipidomic dysregulation within the lung parenchyma following whole-thorax lung irradiation: Markers of injury, inflammation and fibrosis detected by MALDI-MSI

**DOI:** 10.1038/s41598-017-10396-w

**Published:** 2017-09-04

**Authors:** Claire L. Carter, Jace W. Jones, Ann M. Farese, Thomas J. MacVittie, Maureen A. Kane

**Affiliations:** 1 University of Maryland, School of Pharmacy, Department of Pharmaceutical Sciences, 21201 Baltimore, MD USA; 2 University of Maryland, School of Medicine, Department of Radiation Oncology, 21201 Baltimore, MD USA

## Abstract

Radiation-induced lung injury (RILI) is a delayed effect of acute radiation exposure that can limit curative cancer treatment therapies and cause lethality following high-dose whole-thorax lung irradiation (WTLI). To date, the exact mechanisms of injury development following insult remain ill-defined and there are no FDA approved pharmaceutical agents or medical countermeasures. Traditionally, RILI development is considered as three phases, the clinically latent period, the intermediate acute pneumonitis phase and the later fibrotic stage. Utilizing matrix-assisted laser desorption ionization mass spectrometry imaging, we identified a number of lipids that were reflective of disease state or injury. Lipids play central roles in metabolism and cell signaling, and thus reflect the phenotype of the tissue environment, making these molecules pivotal biomarkers in many disease processes. We detected decreases in specific surfactant lipids irrespective of the different pathologies that presented within each sample at 180 days post whole-thorax lung irradiation. We also detected regional increases in ether-linked phospholipids that are the precursors of PAF, and global decreases in lipids that were reflective of severe fibrosis. Taken together our results provide panels of lipids that can differentiate between naïve and irradiated samples, as well as providing potential markers of inflammation and fibrosis.

## Introduction

The pulmonary system is radiosensitive, and radiation-induced lung injury (RILI) is a delayed effect of acute radiation exposure (DEARE)^1–3^. Despite decades of research, RILI remains a dose-limiting complication that can inhibit the administration of curative cancer treatment regimens. Advancements in the precision and delivery of radiation therapy has greatly reduced the incidence of RILI following treatment for thoracic malignancies to ~10–15%, for those presenting with clinical manifestations, although follow-up imaging studies have shown the occurrences of sub-clinical radiation alterations within the lung can be as high as 50%^3–5^. Unlike the radiation dose delivered for the treatment of thoracic malignancies, a radiological accident or nuclear terrorist attack would involve total- or partial-body irradiation (TBI and PBI respectively) with a significant dose being delivered to the whole lung or most of the whole lung. Following such an incident, those that survive the potentially lethal acute radiation syndromes (ARS) of the gastrointestinal (GI-ARS)^6, 7^ and hematopoietic (H-ARS)^8, 9^ systems, often succumb to the delayed pulmonary injury^2, 10^. The dose-response curve for lung lethality following a single dose of radiation has a similar threshold level in humans and the nonhuman primate (NHP) model used in this study. The dose-dependent pulmonary toxicity and lethality increases sharply with doses above 8 Gy, such that an increase of 1 Gy shifts the lethality by almost 50%, resulting in a steep dose-response relationship^2, 11, 12^. To date, there are no FDA-approved treatment regimens or medical countermeasures (MCM) for mitigation of RILI, and the exact mechanisms underlying the developmental process of the complex pulmonary injury that follows radiation insult remains ill-defined.

The histopathological changes of RILI have been well-documented^13, 14^. There is a clinically latent period whereby the patient is asymptomatic, during this time the development of abnormalities to the alveolar epithelium and the vascular network occur. Endothelial and type 1 alveolar epithelial cells (AEC1) undergo apoptosis, there are abnormalities in the shape and size of laminar bodies in type 2 alveolar epithelial cells (AEC2), capillary permeability is increased, interstitial edema is evident, along with increased immune cell populations^13, 15^. This clinically latent period is followed by the intermediate phase, characterized as acute pneumonitis. This stage of disease progression is clinically evident 2–4 months following radiation insult and it can persist for weeks, or occur in cycles for months. Histologically, the alveoli are flooded with mononuclear cells, neutrophils, macrophages, multinucleated giant cells (MGC), edema and proteinaceous material. There is hyperproliferation of AEC2, which are irregular in shape and size. The late phase of RILI follows the intermediate phase and is characterized by lung fibrosis and develops ~6 months post irradiation for high dose exposures and months to years for lower dose exposures^13, 14^. During this phase of RILI there is capillary collapse and reduced permeability, thickening of the basement membrane, loss of entire capillary segments, activated fibroblasts and myofibroblasts, excessive secretion of collagen, and interstitial thickening^4^. Respiratory failure and death can occur at both the intermediate phase of acute pneumonitis and the later phase of pulmonary fibrosis. The complex interplay that results from damage to the many different cells and systems within the lung, spanning the vasculature, epithelium, immune cells and lymphatic systems, means that it is impossible to isolate one single cell or system as the main crux or driving force responsible for the diverse, cyclical and prolonged exacerbations that occur during RILI^16^. Biochemically, there are a number of cytokines^17–19^ and oxidative stress markers^20, 21^ that have been detected at various stages during the development of RILI, yet to date, the exact mechanisms underlying the development and persistence of pneumonitis and the progression to the irreversible condition of fibrosis remains enigmatic.

Our research consortium, the Medical Countermeasures Against Radiological Threats (MCART), has developed animal models that mimic the dose- and time-dependent latency, incidence, severity and progression of radiation-induced injury, spanning the multi-organ syndromes observed as a consequence of TBI, PBI and whole-thorax lung irradiation (WTLI)^2, 6–9, 22–24^. Animal models are vital for the development and approval of MCM under the FDA “animal rule” (FDA-AR), which requires the use of well-defined animal models for the approval of new drugs when human efficacy studies cannot be carried out^24^. We have been applying state-of-the-art analytical techniques to characterize the complexity of radiation-induced injury in an effort to identify and validate diagnostic and prognostic biological markers of organ- or tissue-specific radiation injury^25–29^. Once validated, these biomarkers can be used in pivotal efficacy studies during MCM product development. Our investigations predominantly focus on lipidomic and metabolomic studies as these small molecules play central roles in energy metabolism, signaling, and homeostatic regulation, thus reflecting the phenotype of the tissue environment at a given time^30–36^. Lipidomic and metabolomic alterations have been documented in numerous diseases, including malignancies, cardiovascular, neurological and inflammatory diseases^32, 37–41^.

Herein, we utilized matrix-assisted laser desorption/ionization mass spectrometry imaging (MALDI-MSI) to investigate lipidomic alterations within the differing lung pathologies detected at 180 days following high-dose whole-thorax lung irradiation. We identified multiple lipids species that showed alterations regardless of the pathological presentations following RILI, as well as molecules that correlate with inflammation and changes that reflect severe pulmonary fibrosis.

## Results and Discussion

The naïve lung section presented in the study was used as a representative sample that was taken from a larger study of naive lung tissue in which we previously presented a detailed distribution of the type and intensity of lipid species within the conducting and respiratory airways of NHP biopsy samples^42^. For the investigation of fibrosis and interstitial lung disease, lungs need to be inflated prior to analysis, otherwise the collapsed lung architecture can both mask and mimic disease state profiles. We previously developed a MSI compatible fixation-inflation method for the analysis of lung parenchyma and reported the distribution of lipid species detected in positive and negative ion mode from naïve NHP lung biopsy samples^42^. Our previous investigation of lung tissue by MALDI-MSI determined a minimum resolution of 20 µm acquisition was required to investigate the interstitium and thus the interstitial lung disease that occurs following radiation insult. Acquisitions at 20 µm over an entire lung biopsy section can take anywhere from 40–50+ hours depending on the size of the sample, in order to reduce these acquisition times to something more manageable for a MSI study, acquisitions were carried out over 3 different regions of each lung section. This maximized coverage but reduced the long run times associated with acquiring at 20 µm resolution. As the tissue is fixed there is suppression in the formation of potassium adducts that are often observed when analyzing fresh tissue sections by MALDI-MSI. This not only reduces spectral complexity but it also removes the differences in intensity that are often observed between the sodium and potassium adducts when analyzing disease state tissue^43, 44^. Additionally, previous studies by us and others demonstrated that phospholipids that contain a reactive amine in their structures are cross-linked and formalin-fixed in a time-dependent manner similar to that observed for proteins. This reduced spectral complexity and lipid assignment further as phosphatidylethanolamine (PE) lipid species are fixed and therefore not detected^42, 45^.

### Histological Presentation – Inflammation and Fibrosis

**Figure 1:**
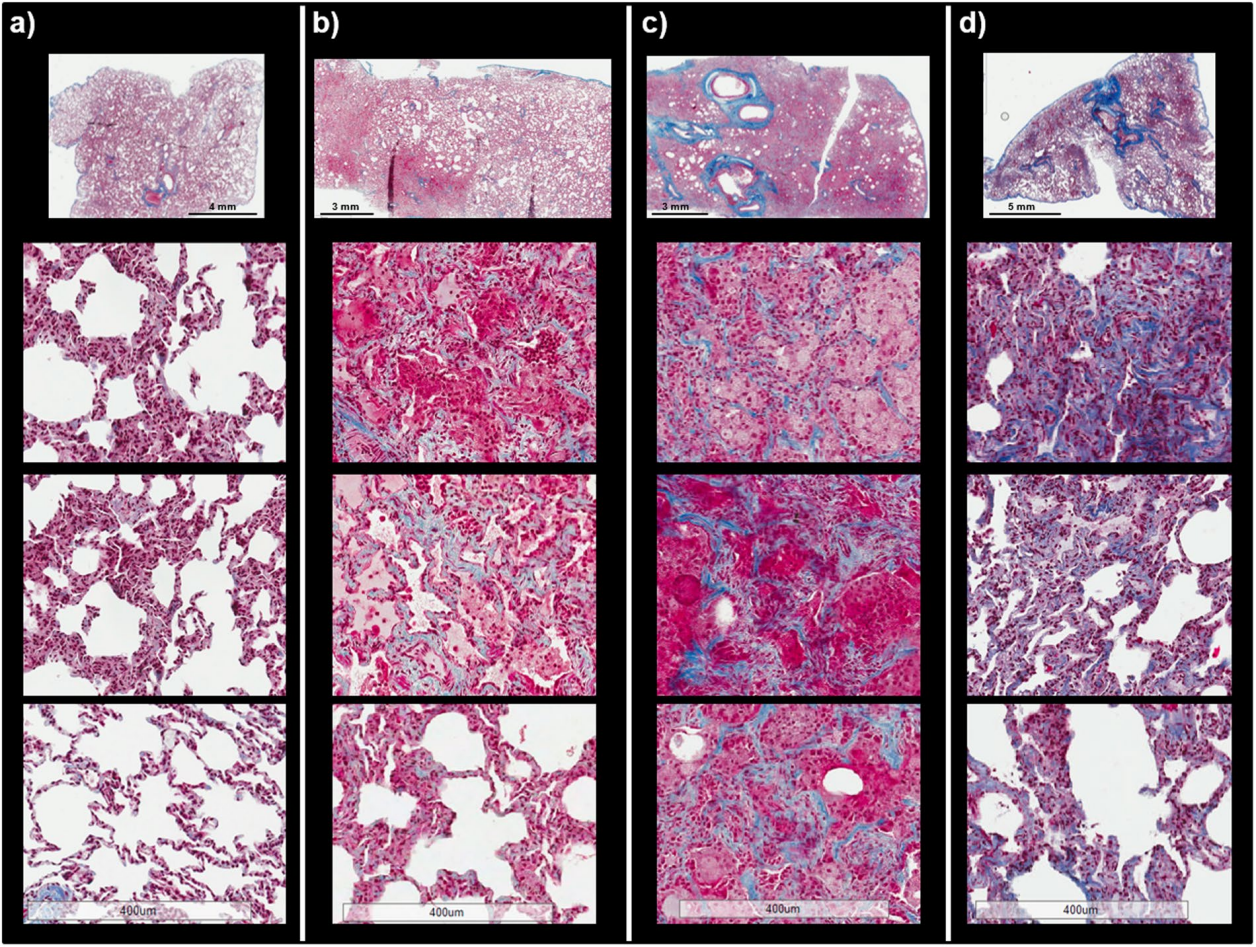
Masson’s trichrome stained sections of lung comparing the naïve and different disease pathologies of the irradiated (IR) animals. Representative Masson’s trichrome stained sections of lung taken from naïve (**a**), IR1 (**b**), IR2 (**c**), and IR3 (**d**). The naïve lung shows clear alveoli space and normal lung architecture. IR1 shows regions of inflammation and edema with normal appearing architecture and mild fibrosis as indicated by the blue stained collagen deposited in the interstitium. The lung section for IR2 is unrecognizable, there is no visible alveoli space, and the lung is flooded with immune infiltrate and alveolar epithelial cell hyperplasia, with severe levels of fibrosis as demonstrated by the blue stained collagen deposited in the interstitium of the whole lung section. IR3 shows regions of disease with some regions of clear alveolar space. Disease pathology again includes edema, immune infiltrate and collagen deposition. The scale bar for the higher magnification images is shown at the bottom of the figure for each sample.

Adjacent lung biopsies to those used for MALDI-MSI were formalin-fixed, processed and paraffin embedded, and stained for collagen deposition using standard clinical protocols. Masson’s trichrome staining was used to detect excessive collagen deposition for the evaluation of fibrosis in each section. Representative Masson’s trichrome stained sections are shown in Fig. 1 for a naïve and the 3 different irradiated (IR) animals discussed in this study. The naïve lung image shown in Fig. 1a shows clear alveolar spaces with no signs of disease state. It should be noted that some of the higher magnification images appear to show thickened interstitium but this is actually a false appearance due to the lungs not being inflated prior to fixation, thus the appearance is from collapsed lung. The 3 different irradiated animals all show varying stages of disease state, as can be seen by comparing their representative stained sections in Fig. 1b-d to the healthy naïve section in Fig. 1a. RILI, as with other pulmonary diseases, presents with variable disease profiles due to the cyclical and sporadic nature of disease development^4, 13^. We added additional trichrome images from 8 different NHP animals as supplementary Figures 1 and 2 to further demonstrate this complexity and heterogeneity. Pockets of normal tissue often reside alongside diseased tissue, and the diseased regions can be highly complex and heterogeneous, thus it is unsurprising these biopsy samples contain varying degrees of pathology. The biopsy taken from the animal designated IR1 (IR = irradiated) shows regions of disease and regions of normal appearing lung with clear alveolar space in the same section, as is often observed following radiation injury^25^. The higher magnification images of the diseased regions show edema and alveolar infiltrate containing mononuclear cells, neutrophils and macrophages. This section also contained regions of mild interstitial fibrosis, as demonstrated by the blue stained collagen deposition in the interstitium. The biopsy taken from the animal designated IR2 shows severe disease progression with no alveolar space visible. This lung displays complete obliteration of the lung parenchyma, all alveolar are filled with infiltrate and there is severe type 2 alveolar epithelial cell (AEC) hyperplasia; the respiratory airways are unrecognizable. Interstitial fibrosis is severe in this section, as demonstrated by the blue-stained collagen deposited throughout the entire section presented in Fig. 1c. The biopsy taken from IR3 shows regions of disease and normal tissue architecture side-by-side, similar to IR1, but with greater disease severity. This section also shows edema, immune infiltrate and hyperplasia, but some alveolar space is visible. There is blue collagen staining in many areas of interstitium throughout this section, the appearance is of moderate interstitial fibrosis.

### Unsupervised multivariate analysis

**Figure 2:**
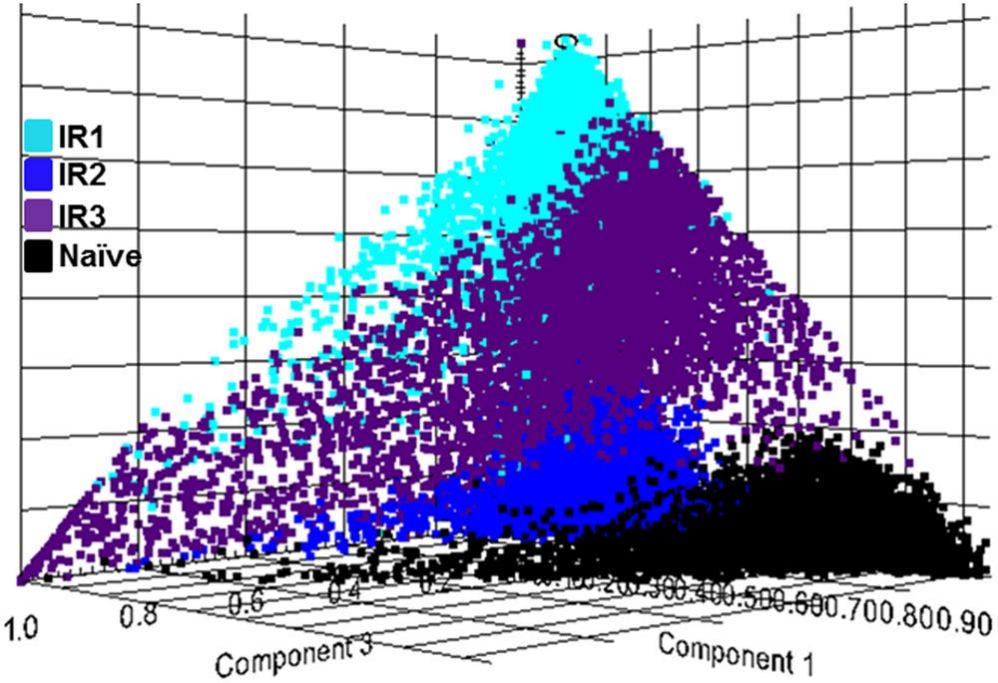
Probabilistic latent semantic analysis (pLSA). 3D scores plot showing the first 3 components from the analysis of ROIs containing 7000 spectra from each of the naïve and the 3 irradiated (IR) samples. The plot shows 4 clear trends for naïve in black, IR1 in light blue, IR2 in dark blue, and IR3 in purple.

Probabilistic latent semantic analysis (pLSA) with deterministic initialization was carried out on the whole data sections and regions of interest (ROI) to observe trends in data across the irradiated samples compared to each other and the naïve sample. ROIs were drawn to compare an even number of spectra and region size across the samples. In all instances the data were separated out into 4 clear trends as shown in the 3D scores plot presented in Fig. 2 and supplementary Figure 3. The results show a clear distinction between the naïve and each of the irradiated samples, the separation into four groups agreed with the data presented from the histological analysis, in that the IR lung biopsies from each animal present with multiple different pathologies and each with varying degrees of severity. The histological evaluation showed that IR1 presented as inflammatory with mild levels of fibrosis, whereas the IR2 lung section is unrecognizable with inflammatory infiltrate, and severe hyperplasia and fibrosis, and IR3 presented with moderate fibrosis and regions of alveolar space still visible. Combined, these results demonstrate that the unsupervised component analysis presented here separated out the different pathological presentations based on their molecular profiles alone, and the groupings correlated with the IR samples having different pathological presentations. The ordination of the IR samples to the naïve dataset also corresponded to the differences in spectral profiles for the three irradiated animals, as shown by the overall average spectra for each dataset presented in supplementary Figure 6. The data shows the spectra profiles with regard to ion formation and ion ratios are more comparable in the naïve sample and IR2 compared to samples IR1 and IR3. This is due changes in lipids signatures caused by the presence of immune cell infiltration and alterations in ion formation caused by dysregulated sodium and potassium pumps, that are commonly perturbed during inflammatory conditions, as previously reported by MSI^43, 44^. Using the data from the pLSA component loadings plot it was possible to identify multiple *m/z* values that differentiate the irradiated lungs from the naïve lungs. These ions were then used to define ROI for further investigation. Discriminating and other non-discriminating *m/z* values were classified further using receiver operating characteristics (ROC) and their area under the curve (AUC) value, as will be presented in the forthcoming sections.

### Injury-associated decreases irrespective of the pathological presentation

**Figure 3:**
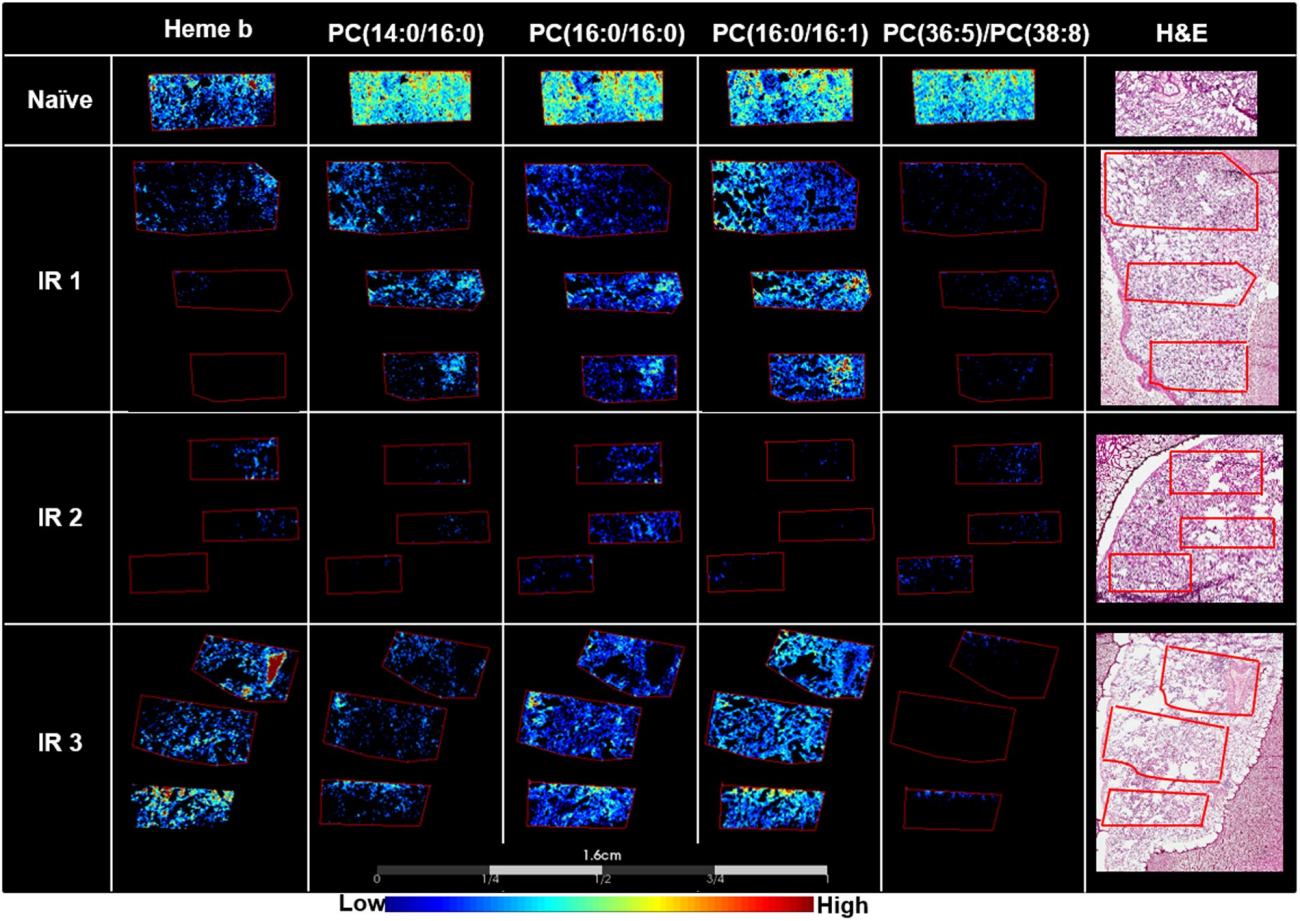
Decreases in heme b and lipids regardless of the pathology. MALDI-MS images showing the decreases in heme b, PC(14:0/16:0), PC(16:0/16:0), PC(16:0/16:1), and PC(36:5)/PC(38:8) in all IR samples compared to the naïve sample. The stained hematoxylin and eosin (H&E) section for each lung sample is shown to the right of the figure.

Results presented herein provided a panel of lipids that were decreased in all sections analyzed to date following radiation insult despite their varying pathological presentations. This is an important finding as these species can be taken forward for further development and validation as biomarkers of radiation-induced lung injury. The most prominent changes were observed with lipids that are the main components of pulmonary surfactant, particularly PC (14:0/16:0), PC (16:0/16:0), PC (16:0/16:1), which showed a significant decrease in intensity across all IR lung samples analyzed irrespective of their pathological presentation. Representative image of these lipids, along with images of heme b, a marker of vasculature, and PC (36:5)/PC(38:8) that showed a significant decrease regardless of pathological presentation is presented in Fig. 3. Higher magnification regions of the H&E sections displayed alongside the MSI images are shown in supplementary Figures 4 and 5, these also demonstrate differences in pathology. Table 1 lists all of the PC species that showed a significant decrease in intensity in the irradiated samples compared to the naïve, each ion is listed with fragmentation data that allowed annotation, or tentative identification. The discriminatory ROC AUC values for each ion detected in the IR sample compared to the representative naïve sample is also shown. Representative spectra from the lung parenchyma of each sample is shown in supplementary Figure 7, data shows a heavy reduction in lipid ions, along with dysregulated profiles as evident by the differences in lipid ratios.

**Table 1:** List of lipid ions that decreased following radiation insult regardless of the pathological presentation within each lung region.

*m/z* Measured	Tentative Lipid Identification	Ion	Fragment Ions	IR1_AUC_	IR2_AUC_	IR3_AUC_
706.5390	PC(30:0)	[M + H] +	184	0.914	0.952	0.894
718.5092	PS(P-32:1)	[M + H] +		0.941	0.911	0.974
728.5222	PC(14:0/16:0)	[M + Na] +	413, 441, 472, 500, 523, 545, 669	0.902	0.956	0.929
732.5612	PC(32:1)	[M + H] +	184	0.770	0.931	0.729
734.5708	PC(16:0/16:0)	[M + H] +	184, 478, 496, 551	0.935	0.964	0.831
754.5389	PC(16:0/16:1)	[M + Na] +	439, 441, 476, 478, 498, 500, 571, 695	0.826	0.943	0.796
756.5525	PC(16:0/16:0)	[M + Na] +	441, 478, 500, 551, 573, 697	0.946	0.968	0.896
792.5883	*PC(P-36:2)/PC(O-36:3)*/PC(P-38:5)/PC(O-38:6)	*[M+Na]+*/[M+H]+		0.725	0.902	0.807
802.5387	*PC(36:5)*/PC(38:8)	*[M+Na]+*/[M+H]+	184	0.918	0.916	0.958

Where multiple lipid species and ion formations are tentatively identified the lipid species as the [M+Na]^+^ ion are shown in italics. An AUC value closer to 1 indicates the *m/z* value is a good candidate marker to discriminate between normal and irradiated samples based on decreases in intensity. Fragment ions are listed as the product ion of the phosphatidylcholine head group or based on neutral losses of the head group and fatty acid side chains with and without sodium and trimethylamine. Measured *m/z* values are from the on-tissue FT profiling experiments and were correlated to the *m/*z values detected during the MALDI-MSI acquisitions.

Phosphatidylcholines form the majority of the lipid rich pulmonary surfactant, which is approximately 90% lipids and 10% proteins, of which dipalmitoylphosphatidylcholine (PC (16:0/16:0)) contributes to over 60% of that PC fraction^46^. Surfactant is a key component of pulmonary physiology and the lipid portion, particularly (PC (16:0/16:0), is important for reducing surface tension at the air-water interface thereby preventing alveolar collapse (atelectasis). A complete loss or significant reduction of these lipids, particularly those observed for (PC (16:0/16:0) will have catastrophic effects on the surface tension of the lungs, leading to possible collapse in these areas. Pulmonary surfactant also plays a key role in protecting the underlying epithelium from toxins and microbes that are inhaled into the lungs from the outside environment^47^. Results presented here, on the loss or reduction in surfactant lipids regardless of the pathological presentation and disease severity, correlate well with previously published data reporting changes in pulmonary surfactant detected from bronchoalveolar lavage (BAL), or alterations in the type 2 alveolar epithelial cells (AEC2) that are responsible for the synthesis and secretion of surfactant, following radiation insult. The first study reporting such changes utilized electron microscopy to demonstrate that pathologically noticeable alterations in the lungs occurred within the AEC2 as early as 1 and 24 hours post IR^15^. Radiation injury to these cells resulted in a reduction in the number of lamellar bodies, which are responsible for the secretion of surfactant by exocytosis, the authors reported these findings were not dose related. Subsequent studies in the 1980’s and early 1990’s showed that surfactant increases in the BAL following radiation injury in both animal and human studies^48–50^. Recently, our laboratory detected a decrease in surfactant lipids, specifically PC(16:0/16:0), in a mouse model of WTLI at 24 h post IR^28^. Linking data from these early time-points to the later 180 day time-points presented here provides evidence for a continued dysfunction in surfactant synthesis and regulation. Thus indicating AEC2 cells do not fully recover from radiation insult and that dysregulation or dysfunctional surfactant cycling persists across the multiple pathologies observed. Data presented here corroborates with findings published for other mechanisms of lung injury. Studies investigating bleomycin-induced lung injury following chemotherapeutic regimens also detected increases in surfactant PCs in BAL, and these findings have been suggested for use as an early marker of bleomycin-induced pulmonary fibrosis^51, 52^. This is a known side effect in up to 10% of patients receiving bleomycin treatment for germ cell tumors or Hodgkin’s lymphoma^53^. A more recent study using a bleomycin mouse model of lung-injury also detected surfactant shedding from AEC2 and correlated this with the appearance of foamy macrophages, linking the cycle of surfactant shedding and dysregulation in AEC2 surfactant reuptake, with the appearance of foamy macrophages, and onset of pulmonary fibrosis^54^. Thus results here correlate with these earlier studies demonstrating that a reduction or loss of surfactant PCs can be used as markers of multiple lung injuries, including radiation-induced pneumonitis and fibrosis. In addition, surfactant lipids have the potential to be developed and validated as early markers of radiation-induced disease progression and could be of use for therapeutic efficacy studies.

Heme b is readily detected by MALDI-MSI and enables facile evaluation of the vasculature in disease state tissues^55^. Detection of heme b at *m/z* 616.2 showed a decrease in the relative intensity across the IR1 and IR2 sections, and a localized or regional decrease in the relative intensity in the IR3 section, as is demonstrated by the regions containing low to no signal for this ion presented in Fig. 3. Damage to endothelial cells and capillary networks is a well-known effect of radiation injury. Capillary networks are thought to replenish depending on the severity of injury, and thus the reduction observed here at the later time point of 180 days post IR is indicative of heavy disease burden and a loss of capillaries^56^. Factors governing endothelial dysfunction and a loss in the capillary network following irradiation are still incompletely understood and were the topic of a recent NIAID workshop^57^.

### Disease specific alterations in the lipid profiles detected

**Figure 4:**
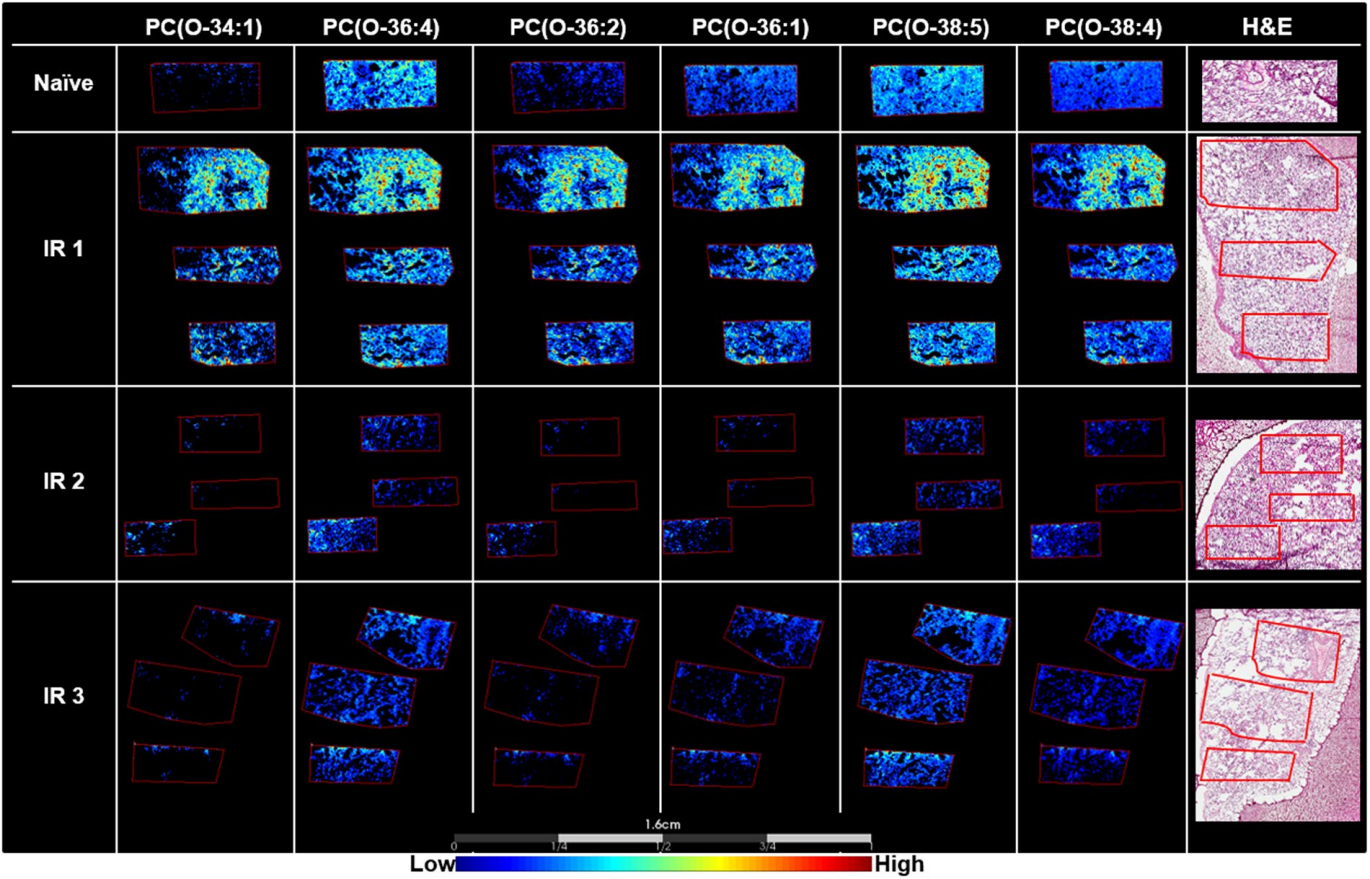
Localized increases in ether lipids are pathology specific. MALDI-MS images showing a region-specific increase in the ether lipids, 1-O-alkyl-2-acyl-*sn*-glycero-3-phosphocholine, in IR1 compared to the naïve, and IR2 and 3 samples. Representative H&E stained sections are shown on the right for each sample.

Several lipid species displayed a localized increase in the lung sample designated IR1, as demonstrated by the regions of high signal intensity for the lipid species presented in Fig. 4. This sample was categorized with less severe disease pathology compared to the samples obtained from 2 other study animals (IR2, IR3). Namely, the histological presentation of IR1 was more inflammatory with regions of clear alveolar space. As the pathology of radiation-induced lung injury vary in disease presentation and severity across these samples it is unsurprising the MSI data also show variations in the molecular profiles detected within each. The lipid species detected in high abundance within this localized region of lung were identified as ether-linked PCs and diacyl PCs containing polyunsaturated fatty acids (PUFAs). A list of ions detected along with their identification based on accurate mass and fragmentation data is presented in Table 2. The ROC AUC discriminatory value for each ion is also presented.

**Table 2:** List of ether-linked PC and PUFA diacyl-PC ions that demonstrated a localized increase following radiation insult.

*m/z* Measured	Tentative Lipid Identification	Ion	Fragment Ions	IR1_AUC_	IR2_AUC_	IR3_AUC_
768.5896	PC(O-34:1)	[M + Na] +		0.147	0.614	0.718
786.6004	PC(36:2)	[M + H] +	184, 502, 506, 524, 603	0.244	0.767	0.587
790.5744	PC(O-36:4)	[M + Na] +		0.369	0.736	0.844
794.6077	PC(O-36:2)	[M + Na] +		0.147	0.651	0.749
796.5900	PC(O-36:1)	[M + Na] +	591, 613, 737	0.257	0.746	0.816
804.5531	PC(36:4)	[M + Na] +	86, 147, 184,	0.217	0.685	0.497
806.5724	PC(36:3)	[M + Na] +	86, 147, 184, 601, 623, 747	0.198	0.701	0.525
808.5891	PC(18:0/18:2)	[M+Na]+	465, 506, 524, 528, 603, 625, 749	0.265	0.763	0.625
816.5870	PC(O-38:5)	[M+Na]+	611, 633, 757	0.295	0.765	0.812
818.5965	PC(O-38:4)	[M+Na]+	613, 635, 759	0.225	0.746	0.850
832.5847	PC(18:0/20:4)	[M+Na]+	469, 489, 528, 548, 627, 649, 773	0.209	0.698	0.523

An AUC value closer to 0 indicates the *m/z* value is a good candidate marker to discriminate between normal and irradiated samples based on increases in relative intensity. Fragment ions are listed as the product ion of the phosphatidylcholine head group or based on neutral losses of the head group. For PC species ions resulting from the neutral loss of the fatty acid side chains with and without sodium and trimethylamine are listed for select ions. Measured *m/z* values are from the on-tissue FT profiling experiments and were correlated to the *m/*z values detected during the MALDI-MSI acquisitions.

Ether-linked PCs are sub-categorized based on the type of bond at the *sn1* position of the glycerol backbone. These can be 1-O-alkyl-2-acyl-*sn*-glycero-3-phosphocholine or 1-O-alk-1′-enyl-2-acyl-*sn*-glycero-3-phosphocholine for ether and vinyl ether linkages, respectively. The vinyl ether linkage groups of phospholipids are more commonly referred to as plasmalogens. Typically, the ether linkage predominate as PCs, whereas the majority of vinyl ether linkages are found in PEs, and this is true for most tissues and cells in the body aside from the heart^58^. Furthermore, ether linked PCs constitute at least half of the PC lipid population in immune cells, including but not limited to, neutrophils, macrophages, monocytes and platelets^59–61^. As the IR1 sample was categorized as inflammatory it is believed the presence of immune cells are the source of the increased detection of these lipids within the lung interstitium of this sample. Fragmentation data obtained identified these lipids as 1-O-alkyl-2-acyl-*sn*-glycero-3-phosphocholine, due to the lack of the unique plasmalogen fragment ion, [M+Na-(182+Na)-R_2_CO_2_H]^+^, that is detected with high abundance in the spectra following dissociation of the alkali adducts of these lipid species^62^. The 1-O-alkyl-2-acyl-*sn*-glycero-3-phosphocholine are important components of immune cells as they are the precursor of a platelet activating factor (PAF, 1-*O*-alkyl-2-acetyl-*sn*-glycero-3-phosphocholine) and other PAF-like bioactive lipids that are produced in these cells via the remodeling pathway^58^. PAF and their bioactive analogues are responsible for a plethora of pathophysiological functions, ranging from the regulation of inflammation and hemostasis to involvement in asthma, allergy, pulmonary distress, vascular permeability, acute bronchoconstriction and sepsis^63–65^. Ether-linked lipids are also a known reservoir for arachidonic acid, the precursor of eicosanoids^59^. There were also several acyl-PCs containing PUFA that showed a significant increase within this area of IR1. PUFA containing PCs can be oxidized into the acyl-PAF analogs and have been shown to be a potent stimulator of the PAF ligands^66^. Additionally, PUFAs can be released from the glycerol backbone of PCs and metabolized into eicosanoids and other important secondary messengers and intracellular modulators.

### Sphingomyelin alterations

**Figure 5:**
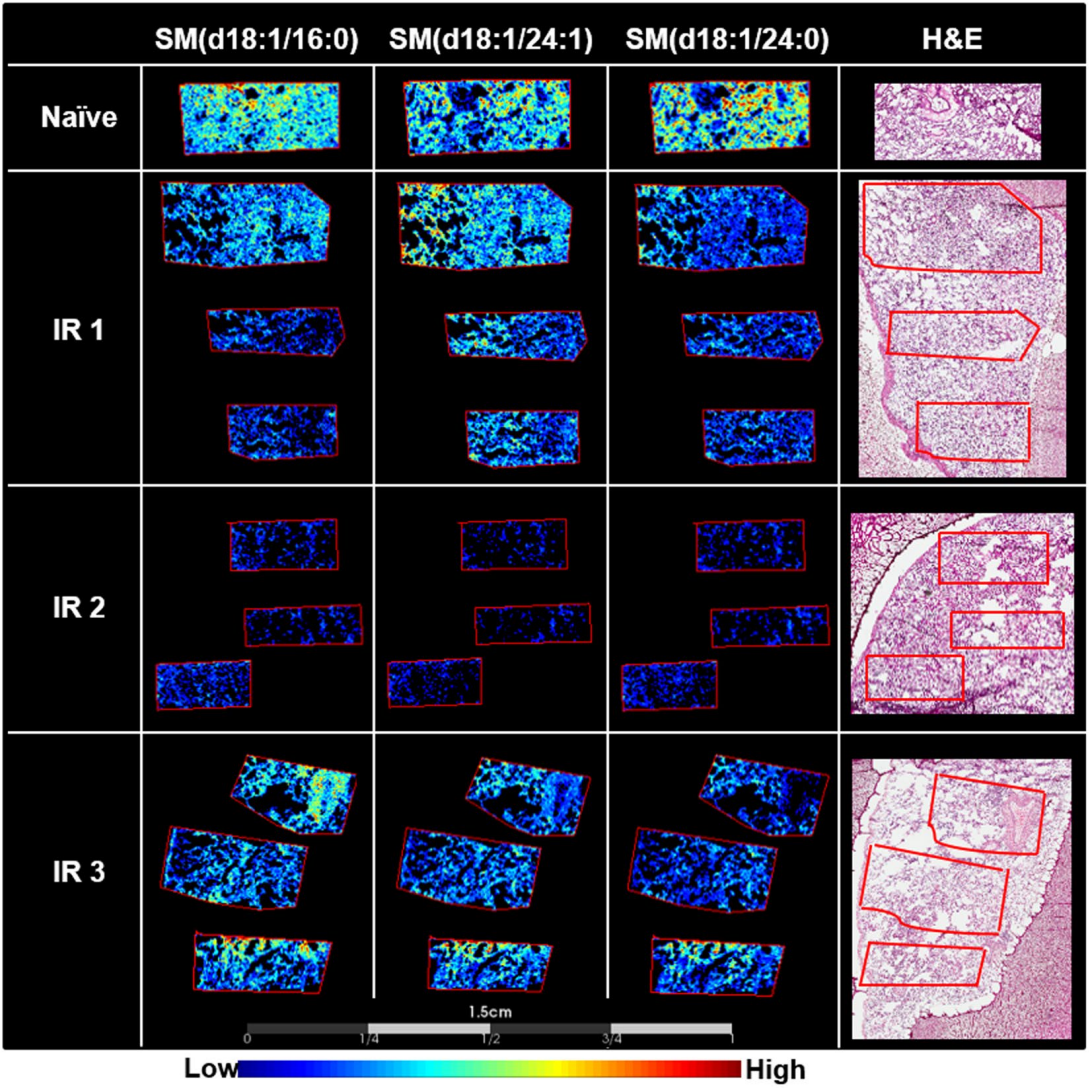
Reduction in the relative intensity of sphingomyelins 180 days post irradiation. MALDI-MS images of sphingomyelin species detected in naïve and irradiated lung samples taken 180 days post radiation. There is a clear decrease in the relative intensity of sphingomyelin species following radiation insult. The decrease is more prominent in the IR2 sample, followed by regional decreases in the IR1 and 3 samples. The stained hematoxylin and eosin (H&E) section for each lung sample is shown to the right of the figure.

A significant decrease in the relative intensity of nearly all sphingomyelin species detected was observed. This decrease appeared to be associated with the level of fibrosis at these later time-points post IR, as the most significant observations were recorded in the severe fibrotic lung parenchyma of the sample taken from IR2, followed by regional decreases in the samples taken from IR3 and IR1. A full list of sphingomyelin species detected is presented in Table 3 and representative images of several of these species are shown in Fig. 5.

**Table 3:** List of sphingomyelin ions detected and their AUC value.

*m/z* Measured	Tentative Lipid Identification	Ion	Fragment Ions	IR1_AUC_	IR2_AUC_	IR3_AUC_
703.5817	SM(d18:1/16:0)	[M + H] +	184	0.671	0.799	0.695
725.5553	SM(d18:1/16:0)	[M + Na] +	86, 147, 184, 542, 666	0.754	0.872	0.724
813.6883	SM(d18:1/24:1)	[M + H] +		0.494	0.805	0.651
815.6998	SM(d18:1/24:0)	[M + H] +	184	0.607	0.808	0.750
833.6654	SM(d18:2/24:1)	[M + Na] +		0.615	0.865	0.632
835.6675	SM(d18:1/24:1)	[M + Na] +	86, 146, 184, 652, 776	0.598	0.849	0.725
837.6860	SM(d18:1/24:0)	[M + Na] +	86, 146, 184, 654, 778	0.735	0.886	0.794

Fragment ions are listed as the product ion of the phosphocholine head group, the neutral loss of the head group, or the neutral loss of the head group and trimethylamine. Where no fragment ions are listed species where tentatively identified based on accurate mass. Measured *m/z* values are from the on-tissue FT profiling experiments and were correlated to the *m/*z values detected during the MALDI-MSI acquisitions.

Sphingolipid dysregulation is a well-known occurrence following insult or injury of any kind to the lung. Elevated levels of ceramide or dysregulation of the ceramide/S1P axis are detected in many pathological conditions, including those observed following radiation insult^67–69^. The hydrolysis of sphingomyelin leading to the production of ceramide following injury or stress to the lung is a well characterized pathway; this sphingomyelin cycle is believed to be responsible for the decreases in the detection of these lipids observed here. Both ceramide levels and the sphingomyelinases responsible for the enzymatic hydrolysis of sphingomyelin to ceramide have been shown to be increased following radiation insult^68, 70^. Results presented here are the first to map the distribution of sphingomyelin species and their dysregulations as it pertains to specific injury or disease severity within lung biopsy samples 180 days post irradiation.

### Severe fibrosis lead to a significant decrease in nearly all lipid species detected

There was a significant decrease in nearly all lipid species detected in one of lung sections analyzed and the decrease correlated with the severity of injury and fibrosis. The biopsy from the animal designated IR2 showed the most drastic lipid changes with a reduction in nearly all lipids identified, as shown by the ROC AUC data presented in Table 4. This table lists the remainder of the lipid species identified through the duration of this study. The reduction or loss in lipids within this lung region would have a profound effect on the local lung physiology and provides evidence of a potential mechanism contributing to the severe impairment of cellular processes and lung function that are not solely attributed to collagen deposition.

**Table 4:** Lipid ions identified through the remainder of the study and their AUC value.

*m/z* Measured	Tentative Lipid Identification	Ion	Fragment Ions	IR1_AUC_	IR2_AUC_	IR3_AUC_
720.5927	PC(O-32:0)	[M + H] +		0.373	0.657	0.914
742.5744	PC(O-32:0)	[M + Na] +	281, 537, 559, 683	0.381	0.659	0.904
758.5686	PC(16:0/18:2)	[M + H] +	478, 496, 502, 575	0.472	0.877	0.641
760.5833	PC(16:0/18:1)	[M + H] +	478, 496, 504, 522, 577	0.425	0.827	0.549
762.6028	PC(34:0)	[M + H]+	184	0.639	0.879	0.744
780.5524	PC(16:0/18:2)	[M+Na]+	441, 465, 478, 500, 502, 524, 597, 721	0.362	0.809	0.624
782.5674	PC(16:0/18:1)	[M+Na]+	441, 467, 478, 500, 504, 526, 599, 723	0.414	0.833	0.568
784.5822	*PC(34:0)*/PC(36:3)	*[M+Na]+*/[M+H]+	86, 147, 184, 725	0.438	0.851	0.642
788.6205	PC(36:1)	[M+H]+	184	0.383	0.812	0.687
810.6024	*PC(36:1)*/PC(38:4)	*[M+Na]+*/[M+H]+	86, 147, 184, 751	0.321	0.812	0.595
830.5696	PC(38:5)	[M+Na]+		0.326	0.775	0.690
834.6038	PC(38:3)	[M+Na]+	86, 147, 629, 651, 775	0.358	0.824	0.724

Fragment ions are listed as the product ion of the phosphatidylcholine head group at m/z 184 or based on the neutral losses of the head group or head group structures (−59 = trimethylamine, −183 = phosphocholine, and −205 = phosphocholine + sodium). Where no fragment ions are listed species where tentatively identified based on accurate mass. Where multiple lipid species and ion formations are tentatively identified the lipid species as the [M + Na]^+^ ion are shown in italics. Measured *m/z* values are from the on-tissue FT profiling experiments and were correlated to the *m/*z values detected during the MALDI-MSI acquisitions.

Combining highly sensitive analytical techniques with well-defined animal models enabled us to investigate local changes in diseased parenchyma, which is difficult in RILI as diseased lung can reside alongside normal appearing lung, and the nature of the disease is highly complex. Using MALDI-MSI to characterize the molecular changes that occur within regions of lung tissue during RILI has provided panels of lipids that can be used to reflect diseases state and have potential to serve as markers for injury in general. Significant differences were observed in lipid species that feed into the ceramide and platelet-activating factor (PAF) pathways, in addition to alterations in surfactant phosphatidylcholines, lipids vital to prevent atelectasis (lung collapse) and infection. These lipids need to be further validated for use as diagnostic and prognostic markers to be taken forward for use in pharmacological efficacy studies. Future studies should delineate the natural history of disease progression to ascertain biomarkers at each stage of development of RILI and before the ongoing insult of pneumonitis and the development of fibrosis. The identification of markers during the latent period of disease progression may be fundamental to unraveling the pathways that lead to the persistent inflammatory insult and aberrant wound healing processes that occur during the development of fibrosis.

## Material and Methods

### Materials

DHB was purchased from Acros (New Jersey, USA). Indium tin oxide-coated glass slides were obtained from Bruker Daltonics (Bruker Daltonics, Bremen, Germany). Analytical grade methanol (MeOH) and water were purchased from Fisher Scientific (Pittsburgh, PA, USA). Masson’s trichrome kit was purchased from Sigma-Aldrich (St. Louis, MO).

### Animals and irradiation

The animals used in this study and the irradiation dosing schedule has been described in detail elsewhere^2^. Briefly, male rhesus macaques (Macaca mulatta, Chinese origin) were irradiated to the whole thorax at a midline tissue dose of 10.74 Gy, this dose was previously determined as an LD(70/180). Animals were monitored twice daily by trained staff at least 6 h apart until the planned end of study [180 (±5) days] or until euthanasia if the animal met the IACUC approved alternate endpoint criteria prior to the end of study. Upon euthanasia lungs were excised and multiple samples were collected frozen in liquid nitrogen and stored at −80 °C until analysis. All animal procedures were conducted in accordance with the NIH guidelines for the care and use of laboratory animals and experiments were performed with prior approval from the University of Maryland Institutional Animal Care and Use Committee (IACUC).

### Histopathology

Lung biopsies residing next to those used for MSI were formalin fixed and paraffin embedded following standard clinical pathology protocols. Sections were taken at 5 µm using a Leica (Leica Biosystems Inc., IL, USA) microtome. Sections were dewaxed, rehydrated and stained with the Masson’s trichrome stain for collagen deposition following the manufacturer’s guidelines.

### Tissue preparation for mass spectrometry

Lung samples from naïve (non-irradiated) and 3 different irradiated (day 180 post-radiation) NHP were formalin-inflated and embedded as previously described^42^. Lung sections were taken at 10 µm using a Microm HM550 cryostat (Themo Fisher Scientific, MA, USA), thaw-mounted onto indium tin oxide-coated conductive glass slides (Bruker Daltonics, Bremen, Germany), and dried in a vacuum desiccator for 20 minutes prior to matrix deposition. DHB at 20 mg/mL in 70% MeOH was deposited over the tissue in 24 cycles using the HTX Sprayer (HTX Technologies, LLC, NC, USA) with the following settings; flow rate was 80 µL/min; 10 psi; 3 mm track spacing; 1333 velocity; the nozzle temperature was maintained at 70 °C and deposition was alternating horizontal and vertical passes. Serial sections were taken on poly-L-lysine coated histopathology slides and stained with hematoxylin and eosin (H&E) for co-registration with MSI datasets, or washed with CHCl_3_:MeOH (1:1) to extract lipids for further identification studies, lipid washes were stored at −20 °C until analysis.

### Mass spectrometry

Multiple datasets were acquired from each animal in a randomized order and reproducibility of the data is shown in supplementary Figures 8 and 9. All imaging experiments were performed using an UltrafleXtreme TOF/TOF mass spectrometer (Bruker Daltonics, Bremen, Germany). The instrument is equipped with a smartbeam II Nd:YAG laser (355 nm) that was operated at sample rate of 1 kHz using the minimum spot size (~30 µm). Acquisitions were carried out in positive ion mode covering the mass range m/z 400–1000. Images were acquired at a spatial resolution of 20 µm summing 200 laser shots per position using the oversampling method^71^. External calibration was performed on a region of lung sections using previously identified lipid masses^42^; internal calibration was performed using the same lipid masses as external calibration. For on-tissue MS/MS analysis LIFT was carried out on selected ions with a mass window of ±1–2 Da. Further fragmentation data was obtained by direct infusion of the lipid washes utilizing collision induced dissociation (CID) a Thermo Orbitrap Elite Velos Pro (Thermo Scientific, San José, CA, USA). Accurate mass measurements for the highly localized or low abundant species that were not readily detected from the lipid washes were acquired in profiling mode using a 12 T FT-ICR mass spectrometer (Bruker Daltonics, Bremen, Germany); acquisition was from the same tissue sections used for imaging. Identification was based on a combination of accurate mass, fragmentation data, and database searches using LipidMaps (www.lipidmaps.org) and Metlin (https://metlin.scripps.edu/).

### Data analysis

Raw data sets were combined into one file and imported into the SCiLS lab software version 2016b (SCiLS Lab, Bremen, Germany). Unsupervised component analysis was carried out using probabilistic latent semantic analysis (pLSA) with deterministic initialization on whole data sets and regions of interest (ROI), ROIs were drawn based on the loading *m/z* ratios and further statistical analysis was carried out to determine the differences in ion type, distribution and intensities between naïve and irradiated samples. For the pLSA component analysis the data was not normalized and the workflow was carried out using all individual spectra. All lipid species identified were grouped according to their type and their discriminating *mass-to-charge ratios (m/z)* with regard to disease-state alterations as determined by receiver operating characteristics (ROC) and the area under the curve (AUC) values.

## Electronic supplementary material

Supplemental Information
