# Supplementary material for: Lipidomic dysregulation within the lung parenchyma following whole-thorax lung irradiation: Markers of injury, inflammation and fibrosis detected by MALDI-MSI
Source: Sci Rep. 2017 Sep 4;7:10343. doi: 10.1038/s41598-017-10396-w (PMC5583385; doi:10.1038/s41598-017-10396-w)
Supplement: Supplementary file 1 — Supplemental Information [file 41598_2017_10396_MOESM1_ESM.pdf]

Lipidomic dysregulation within the lung parenchyma following whole-thorax lung irradiation: Markers of injury, inflammation and fibrosis detected by MALDI-MSI

Claire L. Carter[1], Jace W. Jones[1], Ann M. Farese[2], Thomas J. MacVittie[2], and Maureen A. Kane[1*]

[1]University of Maryland, School of Pharmacy, Department of Pharmaceutical Sciences and [2]University of Maryland, School of Medicine, Department of Radiation Oncology, Baltimore, MD 21201

Correspondence:
Maureen A. Kane
University of Maryland, School of Pharmacy
Department of Pharmaceutical Sciences
20 N. Pine Street, Room N731
Baltimore, MD 21201
Phone: (410) 706-5097
Fax: (410) 706-0886
Email: mkane@rx.umaryland.edu

Supplementary Material

Supplementary Figure list:

Supplementary figure 1. Masson's trichrome images taken from 8 different NHP animals following radiation insult.

Supplementary figure 2. Higher magnification Masson's trichrome images taken from 4 different NHP animals.

Supplementary figure 3. pLSA 3D component plots from A) the whole lung sections and B) the regions of interest (ROIs).

Supplementary figure 4. Higher magnification images of the H&E stained sections used in the MSI study and presented with the MSI data throughout the manuscript.

Supplementary figure 5. Representative higher magnification images of the epithelium taken from the H&E stained sections used in the MSI study and presented with the MSI data throughout the manuscript.

Supplementary figure 6. Overall average spectra from the analysis of the naïve and 3 different irradiated animals presented in this study.

Supplementary figure 7. Representative spectra taken from the lung parenchyma of the naïve and 3 different irradiated animals used in this study.

Supplementary figure 8. Gross images and observations of IR2 is shown in figure A, the stained H&E section is shown in figure B, and images and overall spectra are shown for the 3 technical replicates.

Supplementary figure 9. MALDI-MS image of PC (16:0/16:0) at *m/z* 734.6 and the overall average spectra of this data set. This is a different biopsy taken from IR2 to demonstrate similar pathological profiles from this lung region compared to those presented for IR2.

Supplementary Table 1. Table 1 lists the observed and theoretical m/z, delta m/z and error in ppm for each lipid ion.

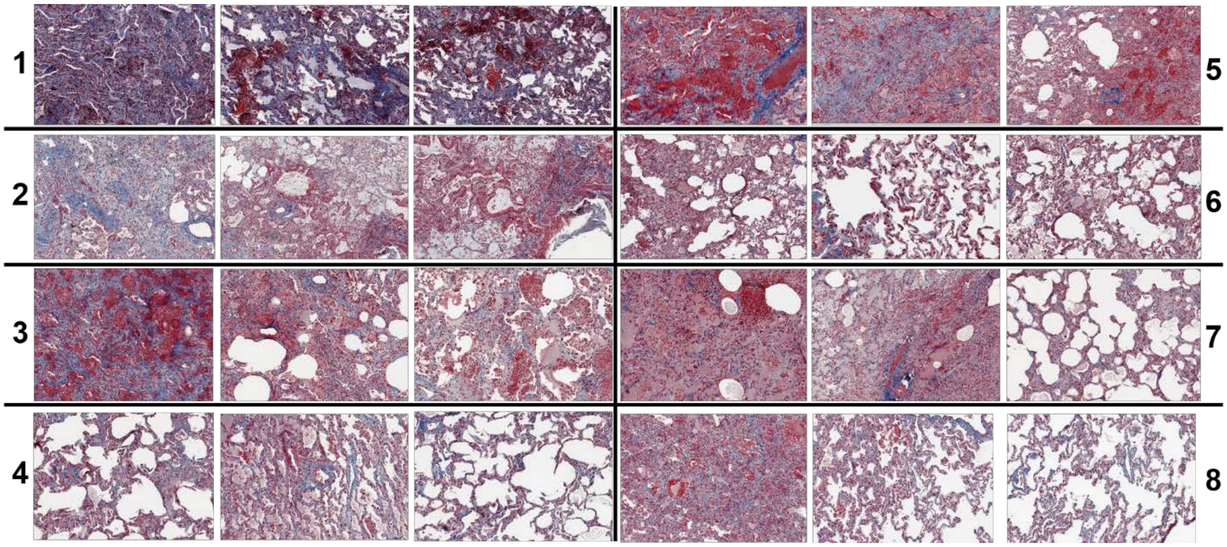

Figure S1. Masson's trichrome images taken from 8 different NHP animals following radiation insult. Each of the 3 images per animal are from the same biopsy section to demonstrate the complexity and heterogeneity within, and across samples, when investigating RILI models that mimic the human condition.

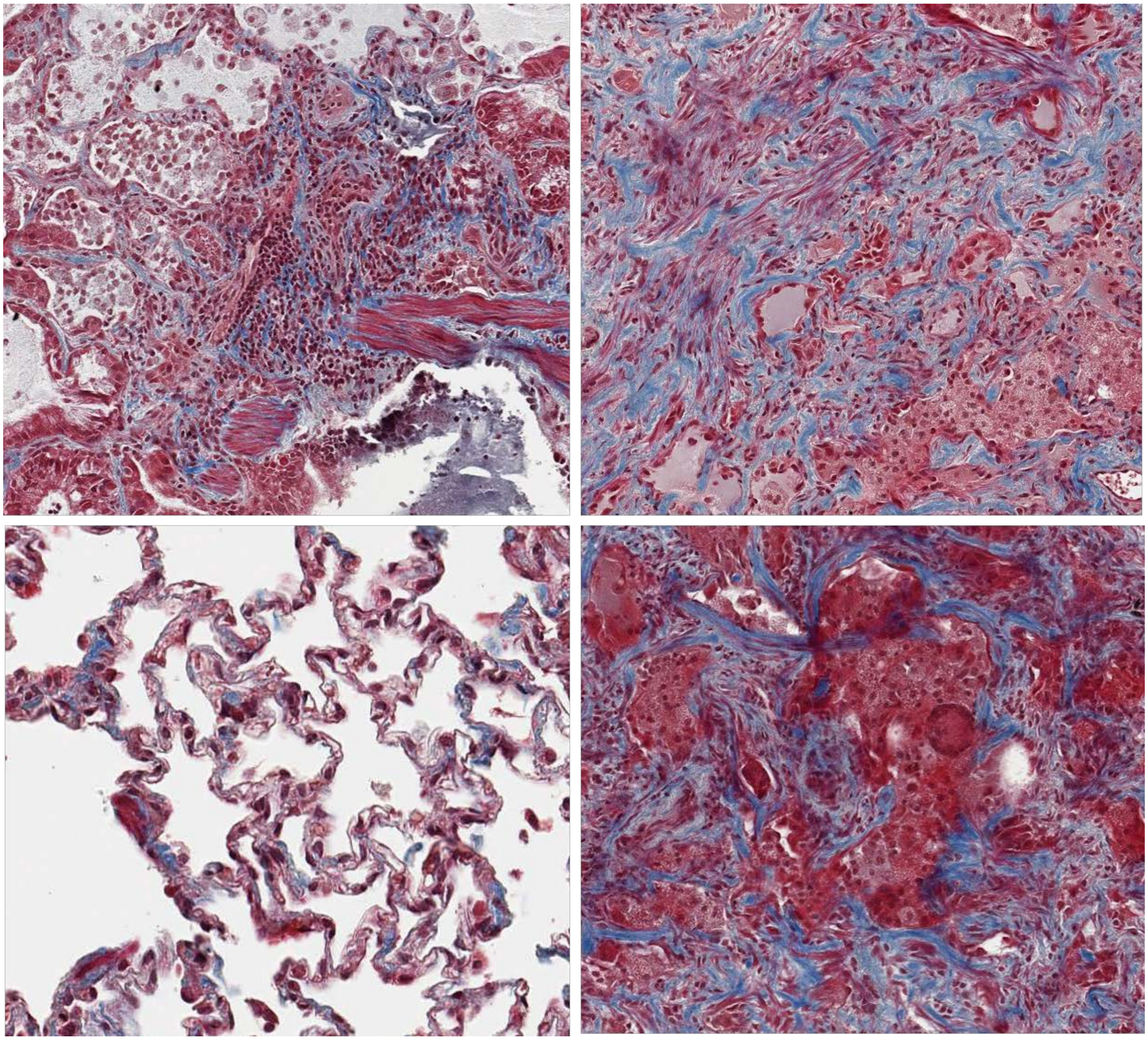

Figure S2. Higher magnification Masson's trichrome images taken from 4 different NHP animals to demonstrate the hypercellular nature, complexity and heterogeneity across samples, when investigating RILI models that mimic the human condition.

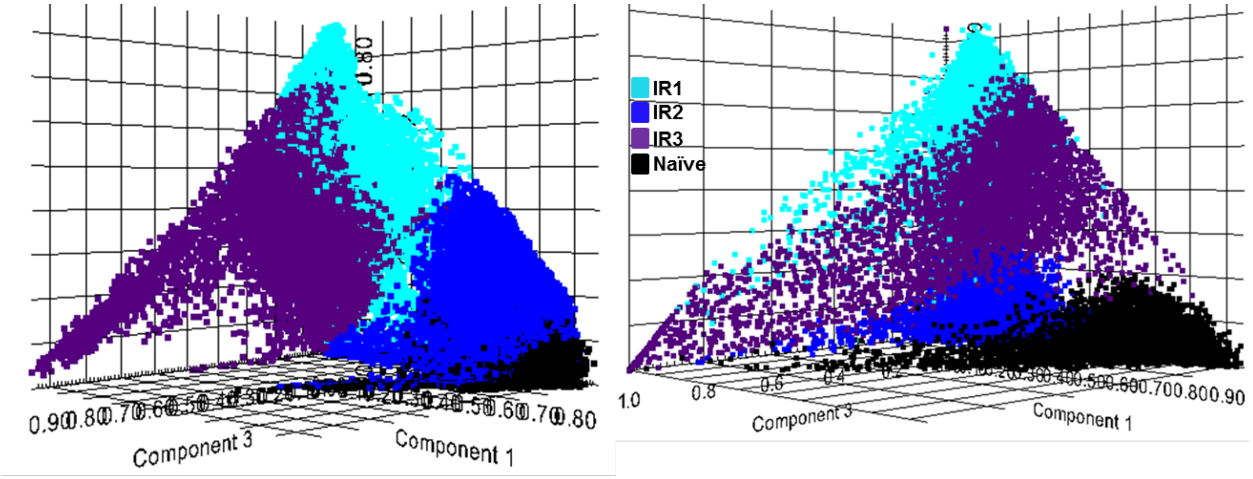

A) whole data sets

B) ROIs

Figure S3. 3D pLSA component plots showing the data are separated into four clear trends for A) the whole data sets and B) regions of interest (ROIs) that compare the same number of spectra in each, ~7000.

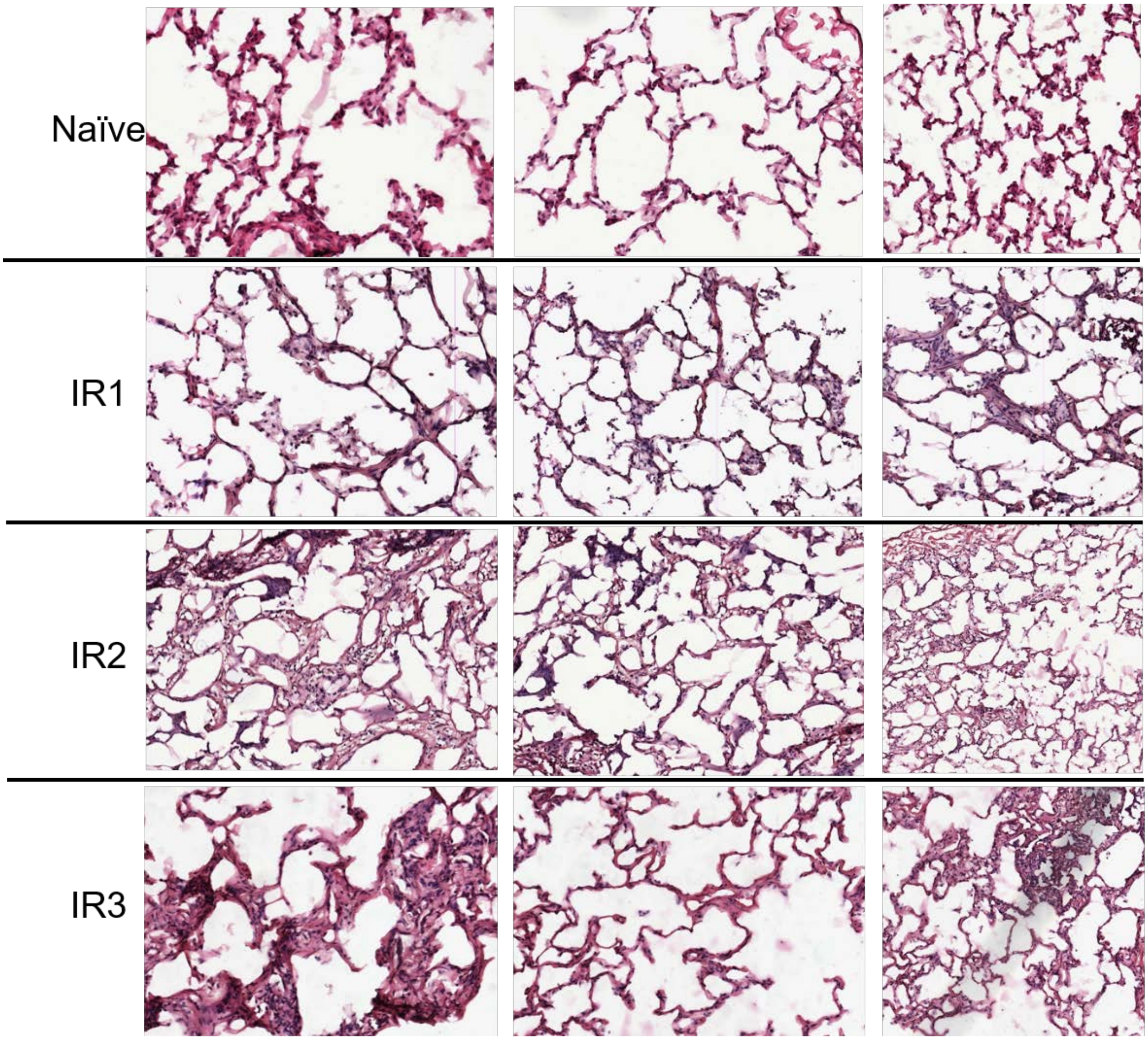

Figure S4. Higher magnification images of the H&E stained sections used in the MSI study and presented with the MSI data throughout the manuscript. The images show abundant nuclei and demonstrate there are no regions void of cells. Infiltrate is still evident in many alveolar regions despite the biopsies being inflated. The differences in pathologies is evident across the samples and compared to the representative naïve sample.

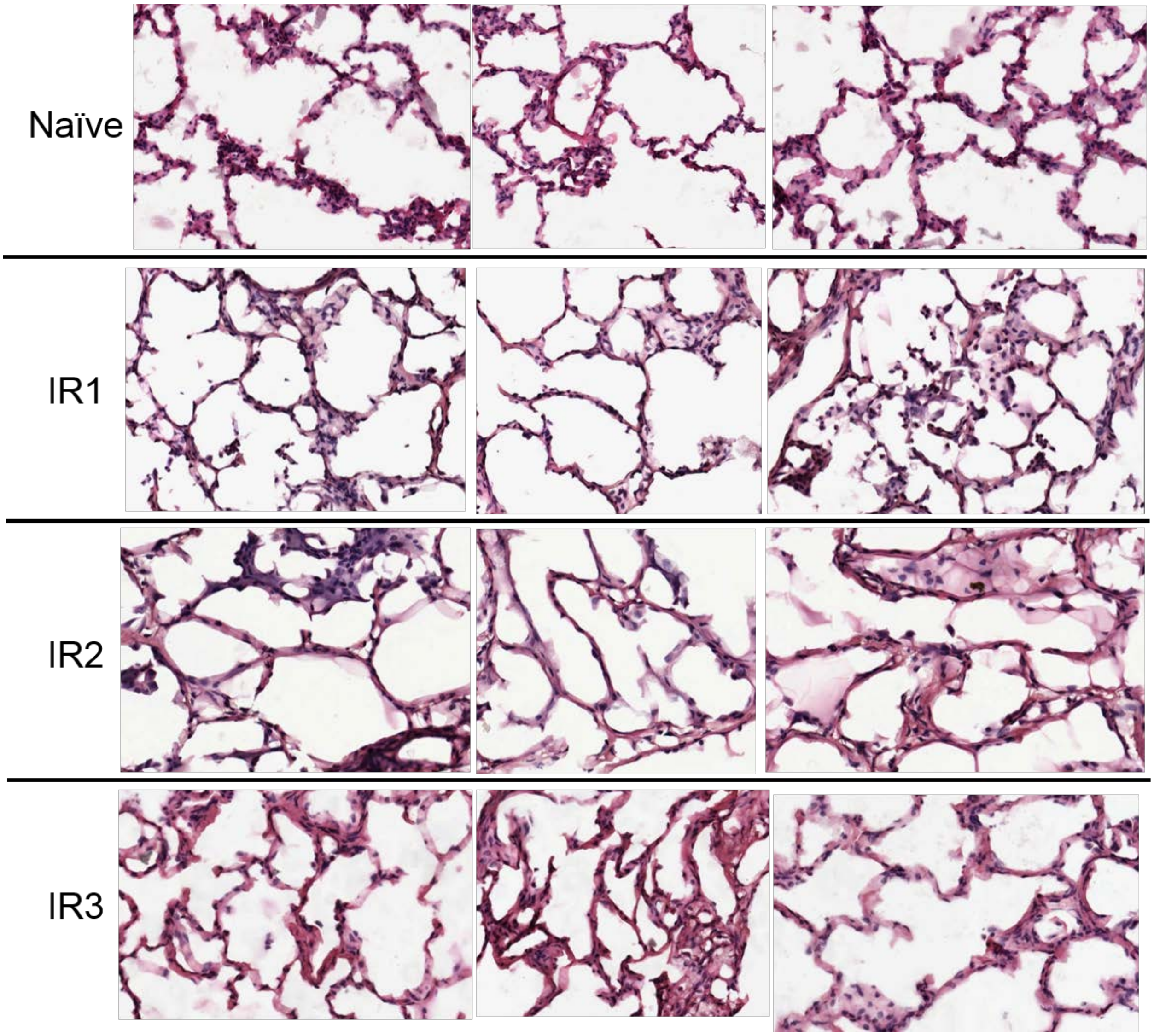

Figure S5. Higher magnification images of the H&E stained sections used in the MSI study and presented with the MSI data throughout the manuscript. The images show abundant nuclei and demonstrate there are no regions void of cells. Infiltrate is still evident in many alveolar regions despite the biopsies being inflated.

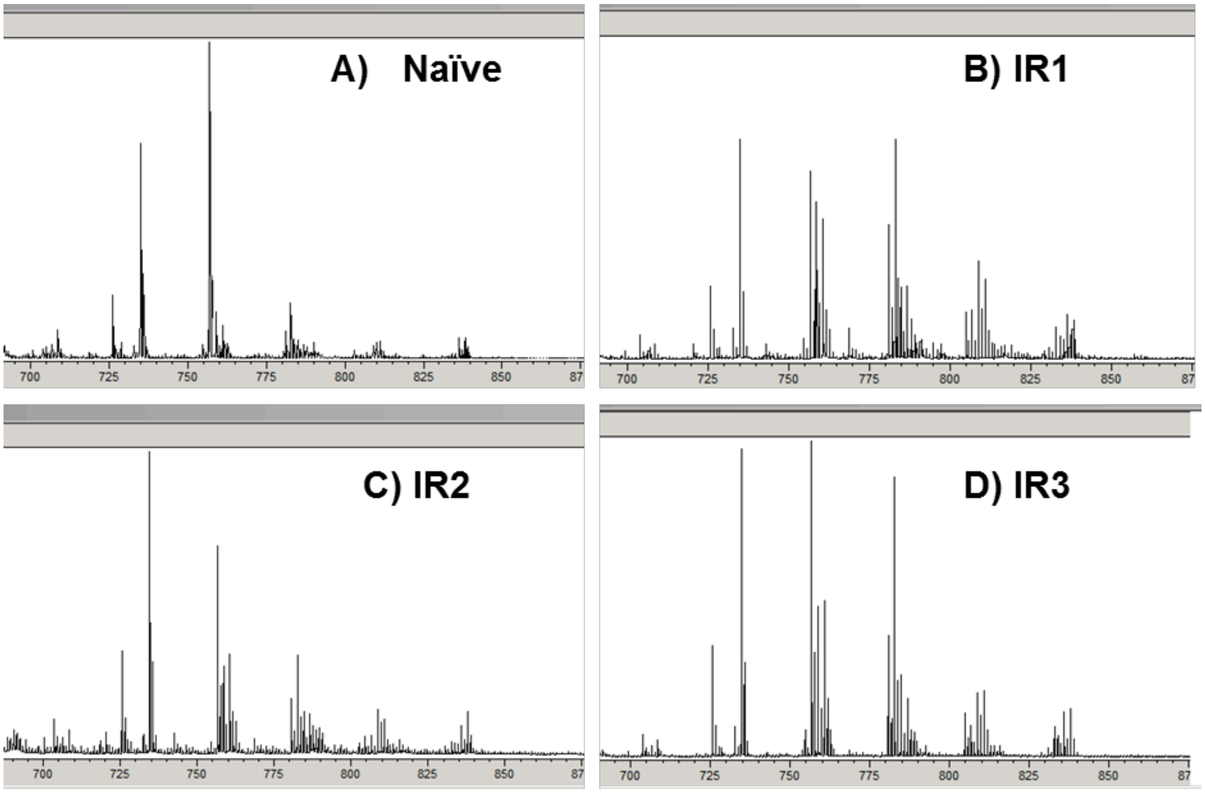

Figure S6. Overall average spectra from the acquisition of the naïve and 3 irradiated samples. The naïve spectrum shows the predominant ion in the lung parenchyma is due to the abundance of the surfactant lipid, PC (16:0/16:0), at *m/z* 734.6 and 756.6. The 3 irradiated sample all show evidence of Lipidomic dysregulation in the lung parenchyma, as evident by the differences in lipid ratios compared to the naïve spectrum.

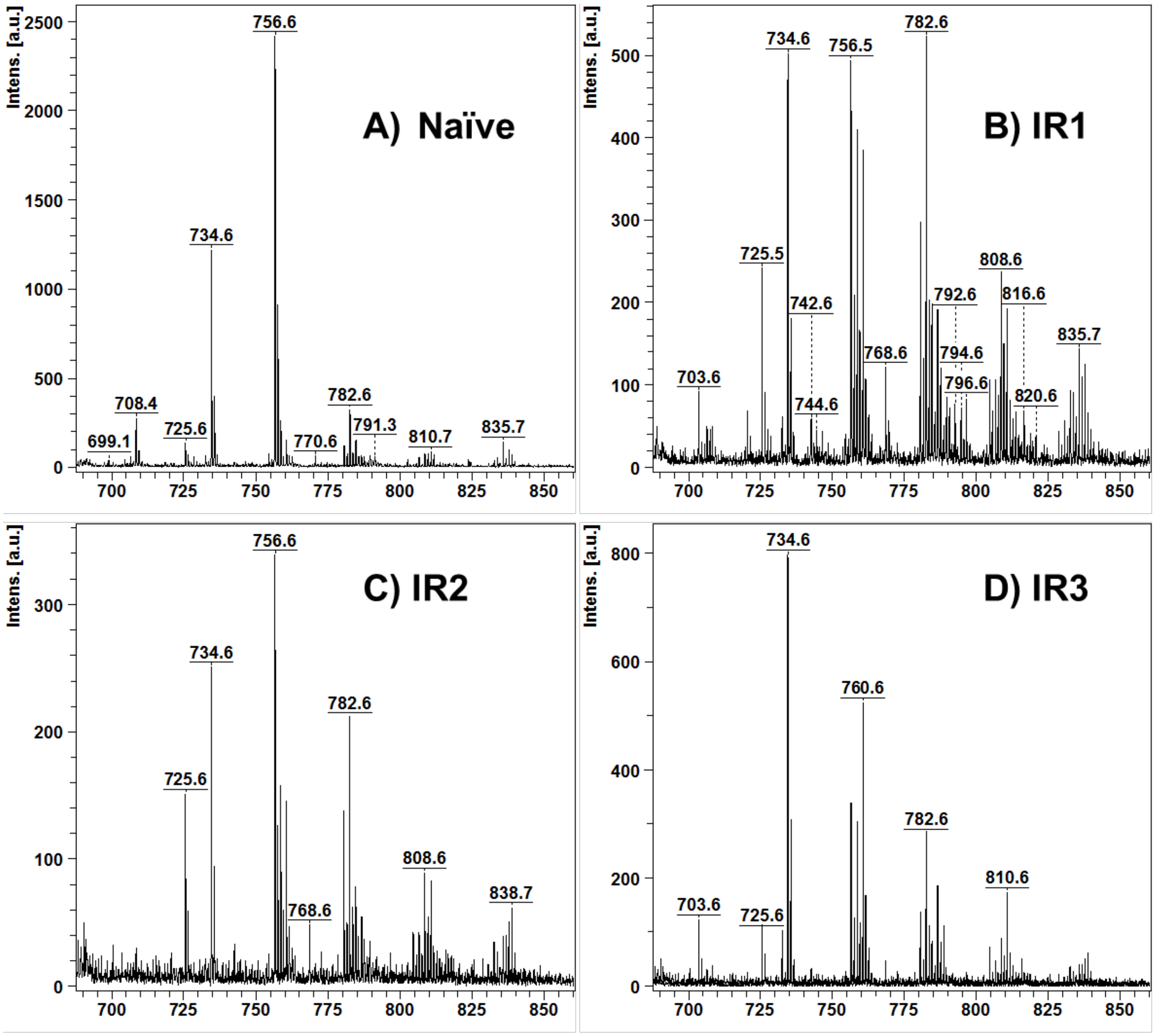

Figure S7. Representative spectra taken from the lung parenchyma of the naïve and 3 different irradiated samples. The dysregulated lipidomic profiles are evident in the 3 different irradiated samples compared to the naïve sample. The decrease in the abundance of the major surfactant lipid, PC (16:0/16:0), at *m/z* 734.6 and 756.6 for the $[M+H]^+$ and the $[M+Na]^+$ ions is clearly demonstrated when comparing IR1, 2 and 3 to the naïve spectrum. The lipidomic ratio is also different thus providing further evidence for dysregulated lipid profiles in the lung parenchyma of the irradiated samples.

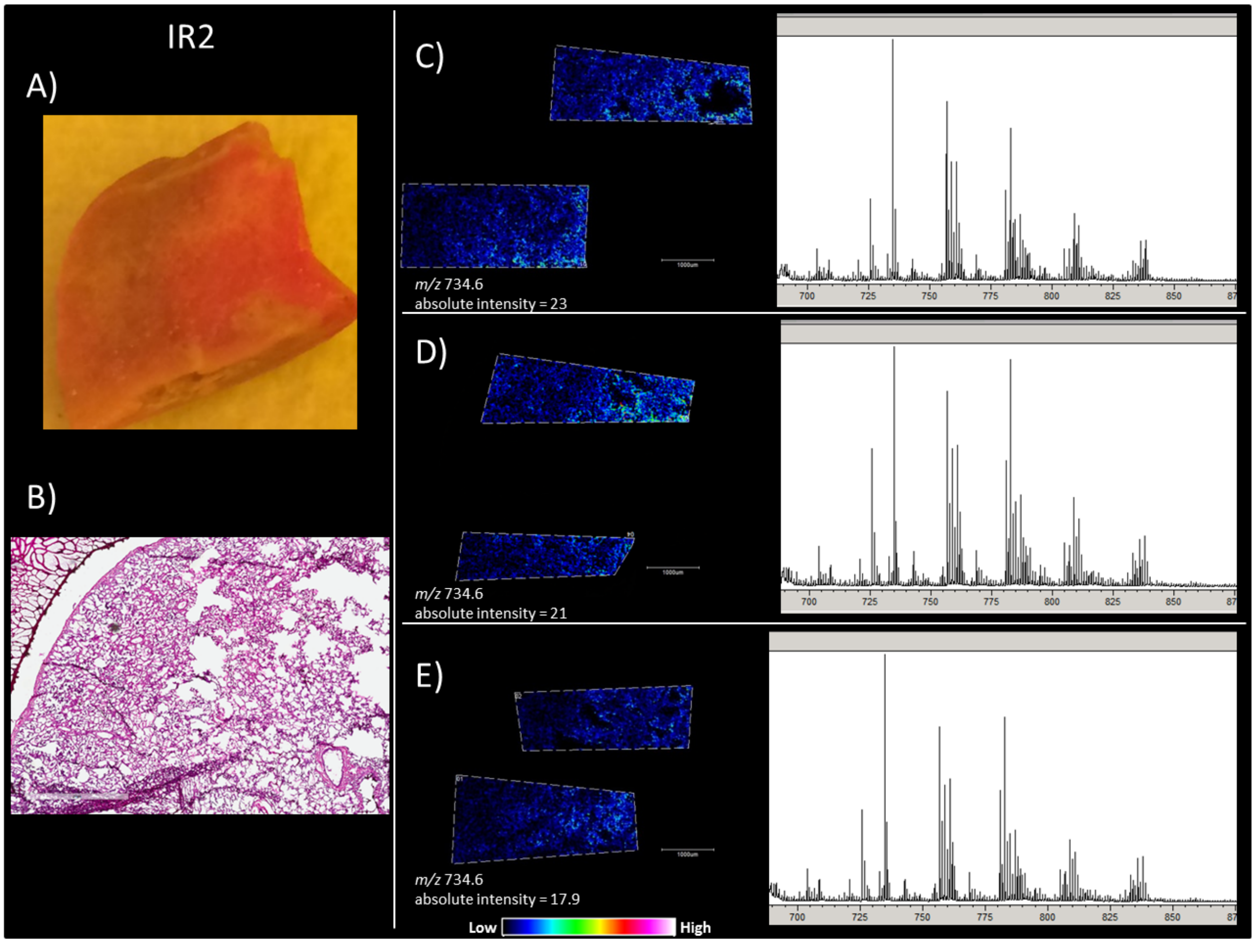

Figure S8. The gross image of the biopsy sample, IR2, is presented in figure S4A. Gross observation shows this lung has brown discoloration along the left and the top and bottom regions. This corresponds with dense staining and consolidation on histology, S4B. The MALDI-MS images of *m/z* 734.6, along with the overall average spectra for 3 technical replicates of IR2, are shown in S4C-D. The images and spectra demonstrate the reproducibility in ion intensity and spectral ratios for the 3 replicates.

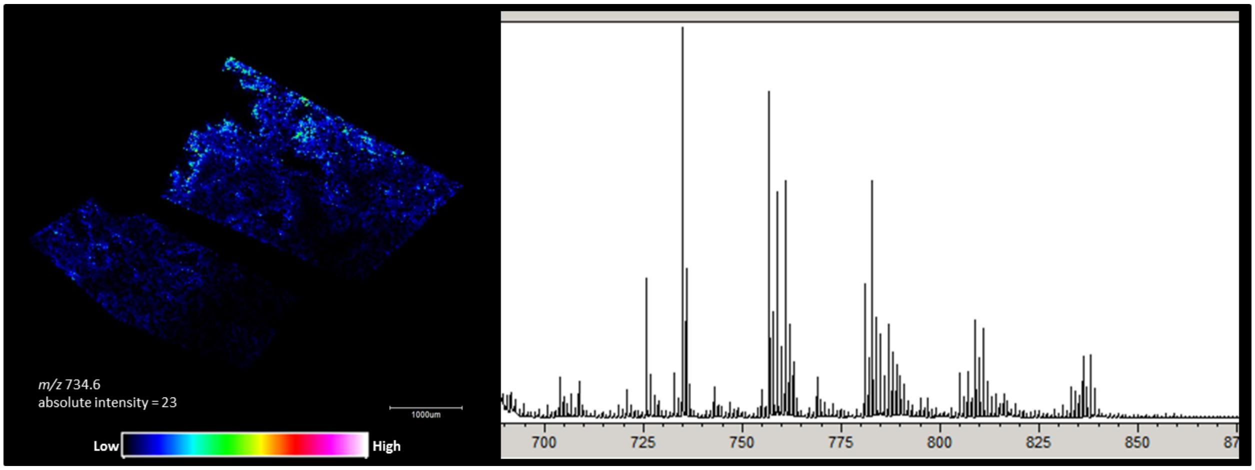

Figure S9. MALDI-MSI image of *m/z* 734.6 along with the overall average spectrum from the acquisition of a second biopsy sample taken from the mid-right lobe of IR2.

m/z Measured	*m/z* Theorectical	delta *m/z*	Error ppm	Tentative Lipid Identification	Ion
703.5817	703.5749	0.0068	9.69	SM(d18:1/16:0)	[M+H]+
706.5390	706.5381	0.0009	1.27	PC(30:0)	[M+H]+
718.5092	718.5017	0.0074	10.35	PS(P-32:1)	[M+H]+
720.5927	720.5902	0.0025	3.46	PC(O-32:0)	[M+H]+
725.5553	725.5568	-0.0015	-2.06	SM(d18:1/16:0)	[M+Na]+
728.5222	728.5201	0.0021	2.92	PC(14:0/16:0)	[M+Na]+
732.5612	732.5538	0.0074	10.09	PC(32:1)	[M+H]+
734.5708	734.5694	0.0014	1.85	PC(16:0/16:0)	[M+H]+
742.5744	742.5721	0.0023	3.08	PC(O-32:0)	[M+Na]+
754.5389	754.5357	0.0032	4.23	PC(16:0/16:1)	[M+Na]+
756.5525	756.5514	0.0012	1.54	PC(16:0/16:0)	[M+Na]+
758.5686	758.5694	-0.0009	-1.16	PC(16:0/18:2)	[M+H]+
760.5833	760.5851	-0.0018	-2.41	PC(16:0/18:1)	[M+H]+
762.6028	762.6007	0.0021	2.75	PC(34:0)	[M+H]+
768.5896	768.5878	0.0018	2.37	PC(O-34:1)	[M+Na]+
780.5524	780.5514	0.0010	1.25	PC(16:0/18:2)	[M+Na]+
782.5674	782.5670	0.0004	0.50	PC(16:0/18:1)	[M+Na]+
784.5822	784.5827	-0.0005	-0.58	**PC(34:0)**/PC(36:3)	**[M+Na]+**/[M+H]+
788.6205	788.6164	0.0041	5.16	PC(36:1)	[M+H]+
790.5744	790.5721	0.0023	2.90	PC(O-36:4)	[M+Na]+
794.6077	794.6034	0.0043	5.40	PC(O-36:2)	[M+Na]+
796.5900	796.6191	-0.0290	-36.45	PC(O-36:1)	[M+Na]+
802.5387	802.5357	0.0030	3.71	**PC(36:5)**/PC(38:8)	**[M+Na]+**/[M+H]+
804.5531	804.5514	0.0017	2.14	PC(36:4)	[M+Na]+
806.5724	806.5670	0.0054	6.66	PC(36:3)	[M+Na]+
808.5891	808.5827	0.0064	7.91	PC(18:0/18:2)	[M+Na]+
810.6024	810.5983	0.0041	5.06	**PC(36:1)**/PC(38:4)	**[M+Na]+**/[M+H]+
813.6883	813.6844	0.0038	4.73	SM(d18:1/24:1)	[M+H]+
815.6998	815.7001	-0.0002	-0.28	SM(d18:1/24:0)	[M+H]+
816.5870	816.5878	-0.0008	-0.98	PC(O-38:5)	[M+Na]+
818.5965	818.6034	-0.0069	-8.40	PC(O-38:4)	[M+Na]+
830.5696	830.5670	0.0026	3.10	PC(38:5)	[M+Na]+
832.5847	832.5827	0.0020	2.43	PC(18:0/20:4)	[M+Na]+
834.6038	834.5983	0.0055	6.57	PC(38:3)	[M+Na]+
835.6675	835.6663	0.001144	1.37	SM(d18:1/24:1)	[M+Na]+
837.6860	837.6820	0.004014	4.79	SM(d18:1/24:0)	[M+Na]+

Supplementary Table 1. Table 1 lists the observed and theoretical m/z, delta m/z and error in ppm for each lipid ion.
